# Characterization of a Fatty Acid Amide Hydrolase (FAAH) in Hirudo verbana

**DOI:** 10.21203/rs.3.rs-4271305/v1

**Published:** 2024-04-18

**Authors:** Emily Kabeiseman, Riley T Paulsen, Brian D Burrell

**Affiliations:** University of South Dakota; University of South Dakota; University of South Dakota

**Keywords:** endocannabinoid, anandamide, leech, invertebrate, neuromodulation, synapse

## Abstract

The endocannabinoid system plays a critical role in modulating both peripheral and central nervous system function. Despite being present throughout the animal kingdom, there has been relatively little investigation of the endocannabinoid system beyond the traditional animal model systems. In this study, we report on the identification and characterization of a fatty acid aminohydrolase (FAAH) in the medicinal leech, *Hirudo verbana*. FAAH is the primary enzyme responsible for metabolizing the endocannabinoid signaling molecule arachidonoyl ethanolamide (anandamide or AEA) and therefore plays a critical role in regulating AEA levels in the nervous system. This *Hirudo* FAAH (HirFAAH) is expressed in the leech central nervous system (CNS) and is an orthologue of FAAH-2 observed in vertebrates. Functionally, HirFAAH has serine hydrolase activity based on activity-based protein profiling (ABPP) studies using the fluorophosphonate probe TAMRA-FP. HirFAAH also hydrolyzes arachidonyl 7-amino, 4-methyl coumarin amide (AAMCA), a substrate specific to FAAH. Hydrolase activity during both the ABPP and AAMCA assays was eliminated by mutation at a conserved activity-binding site. Activity was also blocked by the known FAAH inhibitor, URB597. Treatment of *Hirudo* ganglia with URB597 potentiated synapses made by the pressure-sensitive mechanosensory neuron (P cell), mimicking the effects of exogenously applied AEA. The *Hirudo* CNS has been a useful system in which to study properties of endocannabinoid modulation of nociception relevant to vertebrates. Therefore, this characterization of HirFAAH is an important contribution to comparative studies of the endocannabinoid system.

## Introduction

Endocannabinoids are lipid signaling molecules with a broad range of neuromodulatory effects, especially at the synaptic level [1–3]. Functionally, endocannabinoids are involved in physiological and behavioral processes that include, neurodevelopment, inflammation, pain, cognition, control of affect, appetite and feeding, seizures, brain injury, and neurodegenerative diseases [4]. 2-arachydonoyl glycerol (2-AG) and arachidonoyl ethanolamide (anandamide or AEA) are the two most prevalent endocannabinoids. Their actions are mediated by metabotropic CB1 and CB2 receptors, TRPV1 channels, and the orphan G-protein coupled receptor, GPRC55, although other receptors are may also contribute, e.g., peroxisome proliferator antigen receptors (PPAR). Endocannabinoids are unconventional transmitters in that they are not stored in synaptic vesicles, but are instead synthesized “on demand”, i.e., in an activity-dependent manner, and often in postsynaptic neurons. 2-AG synthesis is mediated diacyl glycerol lipase (DAGL) and is degraded primarily by monoacylglycerol lipase (MAGL), but alternative pathways include alpha/beta hydrolase (ABHD) 6 and 12 [5]. AEA synthesis can be mediated by a N-arachidonoyl phosphatidyl ethanol phospholipase D (NAPE-PLD) or by several different multi-enzyme processes [6]. AEA degradation is mediated by fatty acid aminohydrolase (FAAH) to produce arachidonic acid (AA) and ethanolamine [7], however cyclo-oxygenase 2 (COX-2) has recently been described as an alternative AEA metabolism route [8].

Given the role of endocannabinoids in so many neurophysiological processes, there is considerable interest developing cannabinoid-based therapies for a variety of mental health and neurological conditions [4]. However, relatively little progress has been made in translating research of the endocannabinoid system into actual therapies. For example, while there is considerable interest in using either phyto- or endocannabinoid-based therapies to treat chronic pain, there are still no approved treatments, and the International Association for the Study of Pain does not currently endorse cannabinoid-based therapies to treat pain. Greater success in translation of potential cannabinoid-based therapies depends on greater understanding of the basic biology of the endocannabinoid system. This greater understanding would be facilitated by increased use of comparative approaches to identify evolutionarily conserved mechanisms mediating endocannabinoid neurophysiological and neurobehavioral processes. Such comparative approaches have been successful in understanding fundamental processes, such as those related to learning and memory or neurodevelopment [9, 10].

The endocannabinoid system lends itself to comparative study since endocannabinoid transmitters, 2-AG and AEA, are found across vertebrate and invertebrate species [6, 11, 12]. Similar to vertebrates, endocannabinoid signaling in invertebrates has been shown to contribute learning and memory, and nociception, feeding, axon growth and development, and sensory processing [12–18]. Less detail in known about the molecular signaling mechanisms of endocannabinoids within invertebrates. To date, orthologues have been found for DAGL in *Drosophila* and other invertebrates [19], MAGL in *Hirudo verbana* [20], and FAAH in C *elegans* [21]. Endocannabinoid receptors in invertebrates are more complicated. Orthologues to CB1/CB2 receptors were thought to be absent in invertebrates [11, 22], however two metabotropic receptors in C elegans have recently been proposed as CB1/CB2 orthologues [6, 16, 18]. Transient receptor potential (TRP) channels have been observed as potential endocannabinoid receptors in Drosophila and *Hirudo verbana* [15, 23, 24]. Previous work in our laboratory has used the medicinal leech (*Hirudo verbana*) to study endocannabinoid modulation of nociception at the physiological and behavioral level [12, 24–27], including characterizing critical proteins such as MAGL [20]. In this study we identify a gene encoding the *Hirudo* orthologue of FAAH, assessing its biochemical activity, pharmacology, and potential physiological/synaptic role.

## MATERIALS AND METHODS

### Bioinformatics analysis of HirFAAH

A potential sequence for fatty acid amide hydrolase in *Hirudo verbana* (HirFAAH) accession #GGIQ01042388.1) was identified by tBLASTn search of the *H. verbana* Transcriptome Shotgun Assembly (TSA) sequences using human FAAH (accession NP_001432) as the query sequence. The cDNA sequence was translated using ExPaSy software (https://web.expasy.org/translate/) and the deduced amino acid sequence was analyzed using the protein Basic Local Alignment Search Tool (blastp) from the National Center of Biotechnology Information (https://blast.ncbi.nlm.nih.gov/Blast.cgi). The molecular mass of HirFAAH2 was determined using ExPaSy compute tool (https://web.expasy.org/compute_pi/). Comparison of the putative Hirudo FAAH sequence with human FAAH was carried out using the Ident and Sim algorithms from the Sequence Manipulation Suite (https://www.bioinformatics.org/sms2/ident_sim).

### Cloning and site mutation of HirFAAH

All primers used for cloning projects are listed in Table 1. The full-length coding sequence of HirFAAH2 was generated from total RNA using SuperScript III One-Step RT-PCR system with Platinum Taq DNA Polymerase (Thermo Fisher Scientific Inc., Rockford, IL). To generate an expression construct with 3’ eGFP tag the HirFAAH was inserted into pcDNA3.1eGFP (Addgene plasmid #13031; http://n2t.net/addgene:13031;RRID:Addgene_13031). Single point mutation was generated using the GeneTailor Site-Directed Mutagenesis System (Thermo Scientific). All constructs were confirmed by sequencing (Eurofins MWG Operon, Huntsville, AL). Plasmids were transfected into 293HEK cells (ATCC, Manassas, VA) using Lipofectamine 3000 (Thermo Scientific) as previously described by the manufacturer. For Western blots equal amounts of protein were separated on 10% SDS-PAGE gels and transferred to PVDF. Primary antibodies used were mouse anti-alpha tubulin (Abcam, Cambridge, MA), and mouse anti-GFP (B-2) (Santa Cruz Biotechnology, Dallas, TX). Goat anti-mouse IRDye 680LT was used as a secondary antibody (LiCor Biosciences, Lincoln, NE). Images were taken using the Odyssey CLx and processed using the Image Studio version 5.2 (LiCor Biosciences).

### Quantitative reverse transcriptase-PCR

Total RNA was isolated from four leech nerve cords using the Quick-DNA/RNA microprep plus kit (Zymo Research, Irvine, CA) according to the manufacturer’s instructions. The concentration of the RNA was determined with the Nanodrop 2000 (Thermo Scientific). Oligonucleotide primers used for quantitative reverse transcription PCR (qRT-PCR) are listed in Table 1. The qRT-PCR reactions were performed using the Power SYBR Green RNA-to-C_T_ 1-Step Kit (Applied Biosystems) and ABI Prism 7300 thermocycler (Applied Biosystems), according to the manufacturer’s instructions. qRT-PCR experiments were performed in duplicate from three independently isolated RNA samples.

### Indirect immunofluorescence microscopy

293HEK cells were seeded onto 12 mm coverslips (Carolina Biological, Burlington, NC) and transfected using Lipofectamine 3000 according to the manufacturer’s specifications. 48 hours post-transfection the cells were fixed with 4% paraformaldehyde and permeabilized with 0.05% Triton X-100. Coverslips were counterstained with DAPI and mounted onto glass slides with ProLong Gold antifade mounting medium (Thermo Scientific). Slides were viewed on an Olympus BX60 fluorescent microscope using the 60X objective and images were captured with a Nikon DS-QilMc Camera.

### Activity-based protein profiling of serine hydrolases

The fluorophosphonate probe TAMRA-FP (ActivX Fluorophosphonate Probes, Thermo Scientific) was used to analyze HirFAAH membrane fractions by activity-based protein profiling (ABPP) as previously described [20]. Briefly, 100 μg of membrane fraction was pretreated with 2% DMSO or the FAAH inhibitor URB597 (Cayman Chemicals, Ann Arbor, MI) for one hour at room temperature. This was followed by incubation with 2 μM TAMRA-FP for three hours at room temperature. To stop the reaction 4X SDS-loading buffer was added to a final concentration of 1X and the proteins were resolved on a 10% SDS-PAGE gel. Fluorescence images of the gels were taken with a Typhoon imager (GE Healthcare, Pittsburg, PA) and analyzed using the Image Studio version 5.2 software (LiCor Biosciences).

### Preparation of microsome fractions

FAAH activity was measured in microsomes isolated from HEK cells that over-expressed GFP, HirFAAH or HirFAAH-S225A using a fluorogenic substrate assay as previously described [28]. Briefly, HEK-293 cells expressing HirFAAH, HirFAAH_S225A or vector alone were grown for 48 hours after transfection. The cells were washed twice with ice-cold DPBS and were spun down by centrifugation at 2000 rpm for 10 min at 4°C. Cell pellets were snap frozen in liquid nitrogen and stored at −80°C. The cell pellets were thawed on ice, resuspended in microsome buffer containing 50 mM HEPES (pH 7.4), 1 mM EDTA, and Pierce^™^ Protease Inhibitor Mini Tablet, according to manufacturer’s recommendations, and were sonicated five times for 10 sec, resting for 15 sec on ice between each interval. Immediately following sonication, the cell lysate was centrifuged at 12,000 g for 20 min at 4°C. The pellet was saved as the crude membrane fraction and the supernatant was centrifuged at 100,000 g for 45 min in TLA 100.3 rotor in an Optima Max-XP ultracentrifuge (Beckman). The pellet, microsome fraction, was resuspended in microsome buffer without protease inhibitor cocktail by brief, 10 sec, sonication. Protein concentration was determined with Pierce^™^ BCA Assay kit, the samples were aliquoted, snap frozen in liquid nitrogen and stored at −80°C.

### AAMCA hydrolysis by HirFAAH microsomes

The AAMCA (7-amino-4-methyl Coumarin-Arachidonamide) hydrolysis assay was performed in 96-well black plates (Nunc) in a total volume of 100 μl. To begin, 0.5 μg microsomal protein (in 50 μl), prepared as described above, was incubated with or without the designated inhibitor in assay buffer (50 mM HEPES, 1 mM EDTA (pH7.4) and 1.4 mg/ml bovine serum albumin (BSA, 0.1% final concentration)) for 30 min at room temperature. Immediately following the incubation, 1 μM AAMCA (Cayman Chemical)substrate prepared in assay buffer was added to the microsomal protein, the components were shaken for 2 minutes and measured kinetically for 60 min at 37°C on a Perkin Elmer VICTOR Nivo (PerkinElmer, Waltham, MA) microplate reader. Pure AMC (7-amino-4-mthylcoumarin, Cayman Chemical) was used to generate 0, 20, 40, 60, 80, and 100 pmol/well standard curve of AMC standard in assay buffer. Values were corrected for background fluorescence observed from well containing buffer alone.

### Electrophysiology

Electrophysiology experiments were conducted as recently described in Franzen and Burrell (2023). Briefly, 2–3 g *Hirudo verbana* (North America BioPharma, Erie, CO) were kept in artificial pond water (0.5 g/L Instant Ocean Sea Salt, Aquarium Systems) on a 12-hour light/dark cycle in a 15°C incubator. Animals were anesthetized in 15mM MgCl_2_ saline with 5% ethanol at 4°C for thirty minutes. Individual ganglia were dissected and pinned into 35mm Sylgard-lined dishes for electrophysiology experiments under constant perfusion *Hirudo* saline solution (110mM NaCl, 4mM KCl, 1.8mM CaCl_2_, 1mM MgCl_2_, 5mM NaOH, 10 mM glucose and 10mM HEPES; pH = 7.4) with at a rate of ≈ 2 ml/min. Individual neurons in a ganglion were viewed via a stereomicroscope under darkfield illumination. Dual intracellular recordings were made from one of the pressure-sensitive mechanosensory neurons (P cells) and one of its postsynaptic targets the motor neuron-like anterior pagoda (AP) neuron. Both the P and AP cells were identified by their position and characteristic action potential shape [29]. The P-to-AP synapse has been previously identified as monosynaptic, glutamatergic synapses [30]. Current clamp recordings of P and AP cells were made with a bridge amplifier (BA-1S; NPI, Tamm, Germany) and signals digitally converted for analysis using a DigiData 1322 A (Molecular Devices, Sunnyvale, CA). Current injection into the cells was accomplished using a digital stimulator (Multichannel Systems STG1004; Reutlingen, Germany). Twin P cell action potentials were elicited with a 300 msec interval. The first elicited excitatory post synaptic potential (EPSP) was used to measure changes in amplitude between the pre- and post-test. The second EPSP was used to measure the paired-pulse ratio (PPR = 2nd EPSP/1st EPSP) and provide an indication of whether changes in EPSP amplitude have a pre- or postsynaptic loci [31, 32]. This combined EPSP/PPR recording was repeated at 20 second intervals until 5–10 recordings were obtained and the EPSPs averaged to obtain the first EPSP amplitude and the second EPSP amplitude to calculate the PPR. Post-synaptic input resistance (IR) was monitored by delivering a 500ms, 1nA negative current pulse delivered at 20 second intervals (alternated with the EPSP/PPR recordings). The post-synaptic neuron was hyperpolarized to approximately − 70 mV during EPSP and input IR recordings to prevent postsynaptic action potentials which would interfere with EPSP measurements.

Anandamide (AEA), the FAAH inhibitor URB597, and the TRPV inhibitor SB366791 (Tocris) were prepared on the day of the experiment from frozen aliquots (all stock solutions in DMSO). Following pretest EPSP, PPR and IR recordings, drugs were bath-applied via perfusion of the chamber for 15 mins. For vehicle control experiments, ganglia were treated with 0.001% DMSO. This was followed by a 60 min washout period in normal saline and then post-test measurements of the EPSP, PPR and IR. Following the pretest recording, microelectrodes were removed from the P and AP cells to prevent damage due to osmotic stress and re-inserted during the post-test. Percent change of the IR between the pre- and post-test recordings was used to assess the quality of recordings and only experiments < 15% change in input resistance were included for analysis. Changes in synaptic transmission in a given experiment were based on the percent change in the post-test EPSP amplitude relative to the pretest level (i.e., 100* (EPSP_post_/EPSP_pre_). The percent change in PPR between the pre- and post-test records was calculated in the same way.

### 2.9 Statistics

All data are presented as mean ± standard error. Data were analyzed using 2-way and 1-way Analysis of Variance (ANOVA) using Graphpad/Prism. When appropriate Tukey post-hoc comparisons were also carried out.

## RESULTS

### HirFAAH homology and sequence analysis

**Figure 1:**
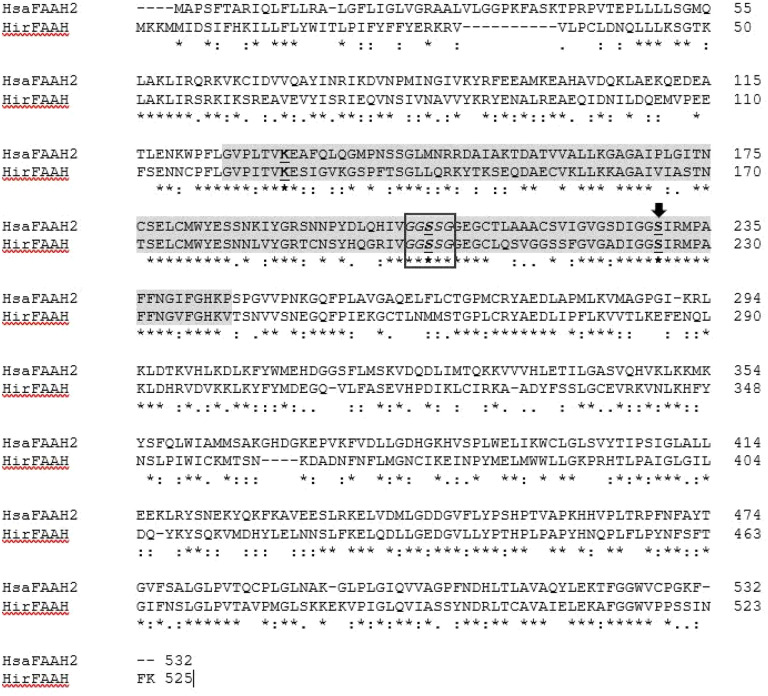
Sequence analysis of FAAH proteins. Clustl O alignments of human FAAH2 (HsaFAAH2) and the predicted FAAH of H. verbana (HirFAAH). Both the HsaFAAH2 active site catalytic triad (K131-S206-S230) and the predicted catalytic triad of HirFAAH (K126-S201-S225A) are in bold and underlined. The catalytic nucleophiles of HsaFAAH2 S230 and HirFAAH S225 are indicated with the black arrow. The GXSCG consensus sequence for serine hydrolases is boxed in black and the conserved amidase signature sequence is highlighted in grey.

**Figure 2:**
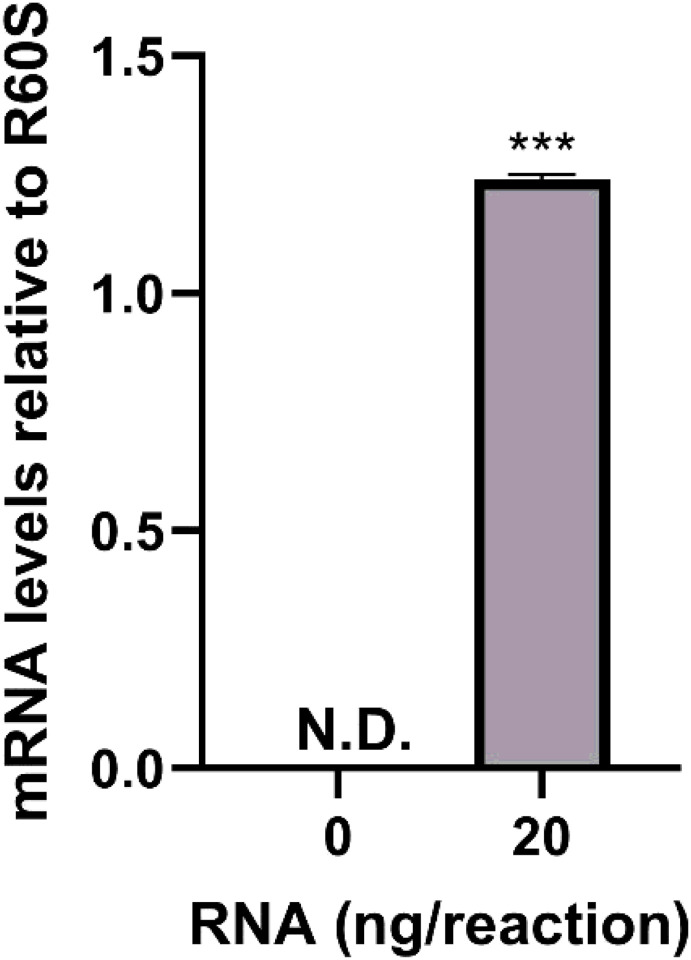
qRT-PCR detection of HirFAAH relative to R60S in the Hirudo CNS. Transcripts of HirFAAH were not detected (N.D.) in the no template control reactions whereas they were detected with 20 ng of total RNA. Mean and standard error of the mean of six independent experiments is reported.

Query of the *H. verbana* TSA using human FAAH amino acid sequence as the query sequence identified the transcribed RNA sequence GGIQ01042388.1. Further analysis of GGIQ01042388.1 resulted in the predicted HirFAAH with a nucleotide length of 1,578 bp, an amino acid length of 525 amino acids and estimated mass of 59.3 kDa. The predicted HirFAAH appears to be more similar to human FAAH-2 (HsaFAAH-2) isoform, based on a 44.1% sequence identity and 59.04% similarity (Fig. 1). The HirFAAH sequence compared to human FAAH-1 revealed 19.19% sequence identity and 33.39% similarity. Consistent with FAAH is other species the HirFAAH sequence possesses a GXSXG motif, a conserved feature in serine hydrolases (black box, Fig. 1). In addition, HirFAAH possesses an amidase signature sequence that exhibits 60.3% sequence identity and 71.1% similarity to HasFAAH2. This amidase sequence contained the serine-serine-lysine catalytic triad (grey highlight and bold/underlined residues, respectively, Fig. 1) and putative catalytic serine (S225) which aligns with the catalytic serine (S230) of HsaFAAH-2 (black arrow, Fig. 2) and are critical for FAAH serine hydrolase activity [33, 34]. Using these sequence data, subsequent qRT-PCR experiments confirmed the expression of HirFAAH in the *Hirudo* central nervous system (CNS; Fig. 2).

**Figure 3:**
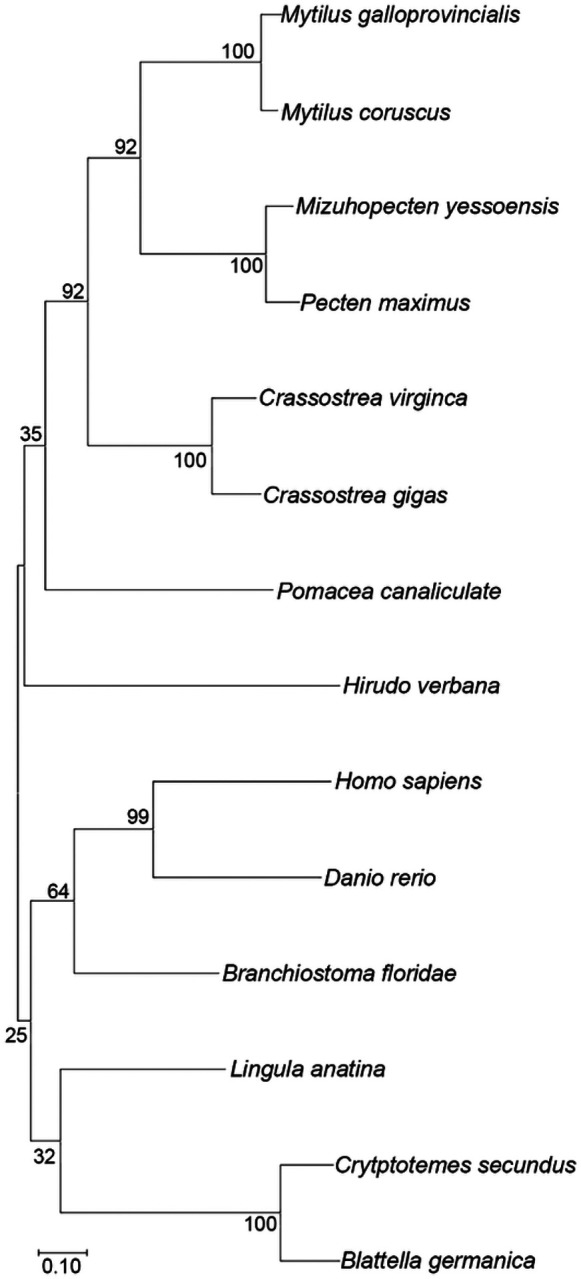
Phylogenetic analysis of HirFAAH. HirFAAH sequences were analyzed in relation to 13 FAAH2/FAAH2-like orthologues by the Maximum Likelihood statistical method, the Jones-Taylor-Thornton (JTT) substitution model for amino acids, and nearest neighbor heuristic method. Confidence intervals for 500 bootstrap trials are provided.

Sequence alignment of HirFAAH were made with FAAH sequences from other species including the mollusk *Mizuhopectin yessoensis*, the brachiopod *Lingula anatina*, arthropod Blattella germanica, and the mammal *Pteropus alecto*. Along and *Homo sapiens* FAAH (hsaFAAH) these show a high degree of homology in the region making up the catalytic triad and at the C-terminus (data not shown). A phylogenetic tree (Fig. 3) of confirmed or provisional FAAH2 orthologues from other species was constructed with HirFAAH using sequences from the Mediterranean mussel, Mytilus galloprovincialis (VDI01341.1), the Eastern oyster, *Crassostrea virginica* (XP_022339113.1), the scallop, *Mizuhopectin yessoensis* (OWF55125.1), the Korean mussel, *Mytilus coruscus* (CAC5390857.1), the Florida lancet, *Branchiostoma floridae* (XP_035686393.1), from the pacific oyster, *Crassostrea gigas* (XP_034331103.1), the termite, *Cryptotermes secundus* (XP_023715933.1), from the great scallop, *Pectin maximus* (XP_033740866.1), the snail, *Pomacea canaliculate* (XP_025099255.1), the German cockroach, *Blattella germanica* (PSN54917.1), the brachiopod, *Lingula anatine* (XP_013399812.1), Zebrafish, Danio rerio (NP_001002700.1), and Homo sapiens (NP_777572.2). Sequences ranged from 67.76–43.76% identity matches to the predicted HirFAAH sequence. The phylogenetic groupings of FAAH2 orthologues were largely as one might expect. The *Hirudo* HirFAAH sequenced grouped with other lophotrochozoan invertebrate species (e.g., mollusks and annelids) and these were distinct from ecdysozoans (e.g., cockroach and termites) and vertebrates (e.g., lancets, zebrafish, and humans). The FAAH2 sequence from the brachiopod *Lingula* appeared to be distinct from all the other phylogenetic groupings despite being a lophotrochozoan.

### Features of HirFAAH expression in HEK-293 cells

**Figure 4:**
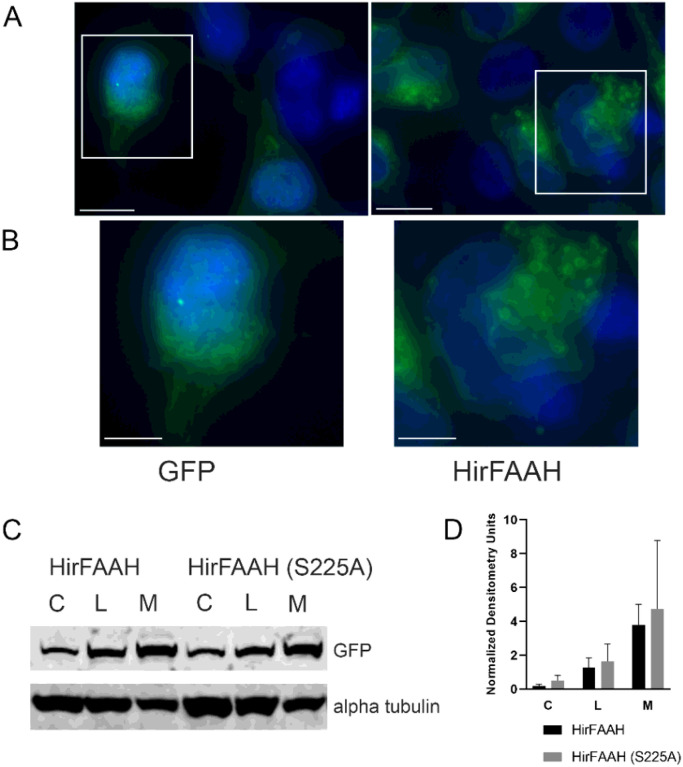
HirFAAH cellular localization. (a) The localization of GFP and HirFAAH in transfected 293HEK cells. Scale bars = 10 μm. (b) Inset from (a) showing the ring structures in the HirFAAH transfected cells that is not observed in the GFP controls. Scale bars = 5 μm. (c) Western blot analysis of whole cell lysate (L), cytoplasmic (C) and membrane (M) fractions of 293HEK HirFAAH and HirFAAH(S225A) expressing lysates. (d) Relative expression of the S225A mutant and wild type enzymes based on the quantification of the GFP protein band normalized to the loading control alpha tubulin.

The cloned HirFAAH was labeled in frame with a C-terminal eGFP tag resulting in a protein 768 amino acids in length and expressed in 293-HEK cells (Fig. 4). Immunofluorescence microscopy indicated the tagged HirFAAH was localized to hollow, ring-like cytoplasmic structures (Fig. 4a, b). This is consistent with immunofluorescence microscopy experiments performed with HsaFAAH2 that also localized to ring-like structures (Fig. 4a, b) [35]. The eGFP tagged HirFAAH protein was enriched in the membrane fractions of transfected 293-HEK cells (Fig. 4B, C), again consistent with the subcellular distribution of other FAAHs [36, 37]. We also tested a version of HirFAAH with a mutation of the active site serine (S225A). This mutation did not prevent expression of HirFAAH in the 293-HEK cells, nor did it disrupt localization to the membrane fraction as seen in wildtype FAAH (Fig. 4c, d).

### Activity-Based Protein Profiling

**Figure 5:**
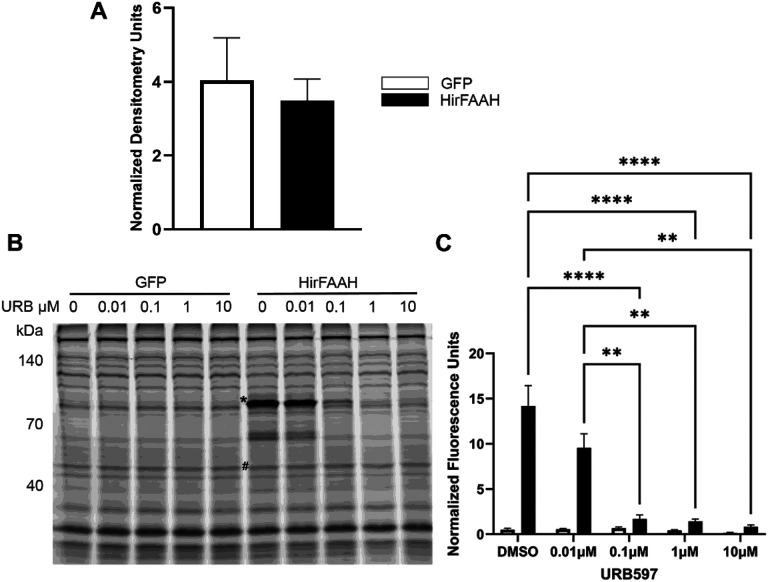
Serine hydrolase activity of HirFAAH. (a) Relative expression of GFP and HirFAAH in membrane fractions based on the quantification of the GFP protein band normalized to the loading control alpha tubulin. (b) Representative fluorescent image of TAMRA-FP^™^ labeled membrane fractions from GFP- and HirFAAH-expressing cells. An asterisk (*) notes serine hydrolase activity at a molecular weight (83 kDa) consistent with FAAH that is not present in the GFP samples. Both GFP- and HirFAAH-containing samples were treated with increasing concetrations of URB597, which decreased serine hydrolase activity at the 83 kDa band. (c) Quantitation of serine hydrolase activity with increasing concentrations of URB597 was measured in the HirFAAH band (*) or 83 kDa region in the GFP samples was normalized to the fluorescence signal from the 55kDa band (#), panel B. Activity is shown as the mean and standard error and signficant decreases in activity were observed at 0.1, 1, and 10 μM URB (****P<0.0001 and ** P<0.01).

The catalytic activity of HirFAAH was assessed by ABPP, which detects functionally active serine hydrolases [34, 38]. Membrane fractions were prepared from 293HEK cells transfected with HirFAAH, HirFAAH(S225A), or GFP vector and the relative expression of each protein was determined via western blot (Fig. 5a). These findings indicated successful transfection and expression for all three proteins. The ActiveX^™^ TAMRA-FP probe labeled an ~ 83 kDa protein in the HirFAAH expressing membrane fractions that corresponded with the expected molecular weight of GFP tagged HirFAAH (Figs. 5b; N = 9). Hydrolase activity at this molecular weight was not present in any of the GFP lanes (Fig. 5b; N = 7). Next, we tested the effects of URB597, a selective, irreversible inhibitor of mammalian FAAH [39], on the serine hydrolase activity of HirFAAH at concentrations 0.01, 0.1, 1 and 10 μM plus a vehicle control (2% DMSO). A 2-way ANOVA was used to assess serine hydrolase activity in samples from HirFAAH-transfected cells vs. those transfected with the GFP-containing vector and the effects of increasing concentrations of URB97 on hydrolase activity. This analysis detected a significant effect of transfection construct with serine hydrolase activity with HirFAAH samples have much greater activity than GFP-only samples (F_1,46_ = 16.84, p < 0.0001). Preincubation of sample with URB597 decreased hydrolase activity in the HirFAAH in a concentration-dependent manner that was not observed in the GFP samples (Fig. 5b, c). This was confirmed by a significant concentration effect (F_4,46_ = 23.16, p < 0.0001) and a significant interaction effect (F_4,46_ = 22.88, p < 0.0001). Post-hoc analysis indicated significant inhibition of HirFAAH activity by 0.1, 1, and 10 μM URB597 (p < 0.0001 for all). No other proteins labeled by the AcivX^™^ TAMRA-FP probe exhibited any obvious sensitivity to URB597.

### AAMCA-based Fluorescence Assay

While the ABPP assay confirms serine hydrolase activity, it does not confirm that HirFAAH actually metabolizes AEA. To address this issue, we used a high-throughput fluorescent screening assay developed by Ramarao et. al. that specifically measures FAAH activity [40]. In this assay, FAAH catalyzes the hydrolysis of a nonfluorescent AAMCA, a substrate that is specific to FAAH, to produce arachidonic acid and the highly uorescent AMC (excitation 355nm/emission 460nm).

**Figure 6:**
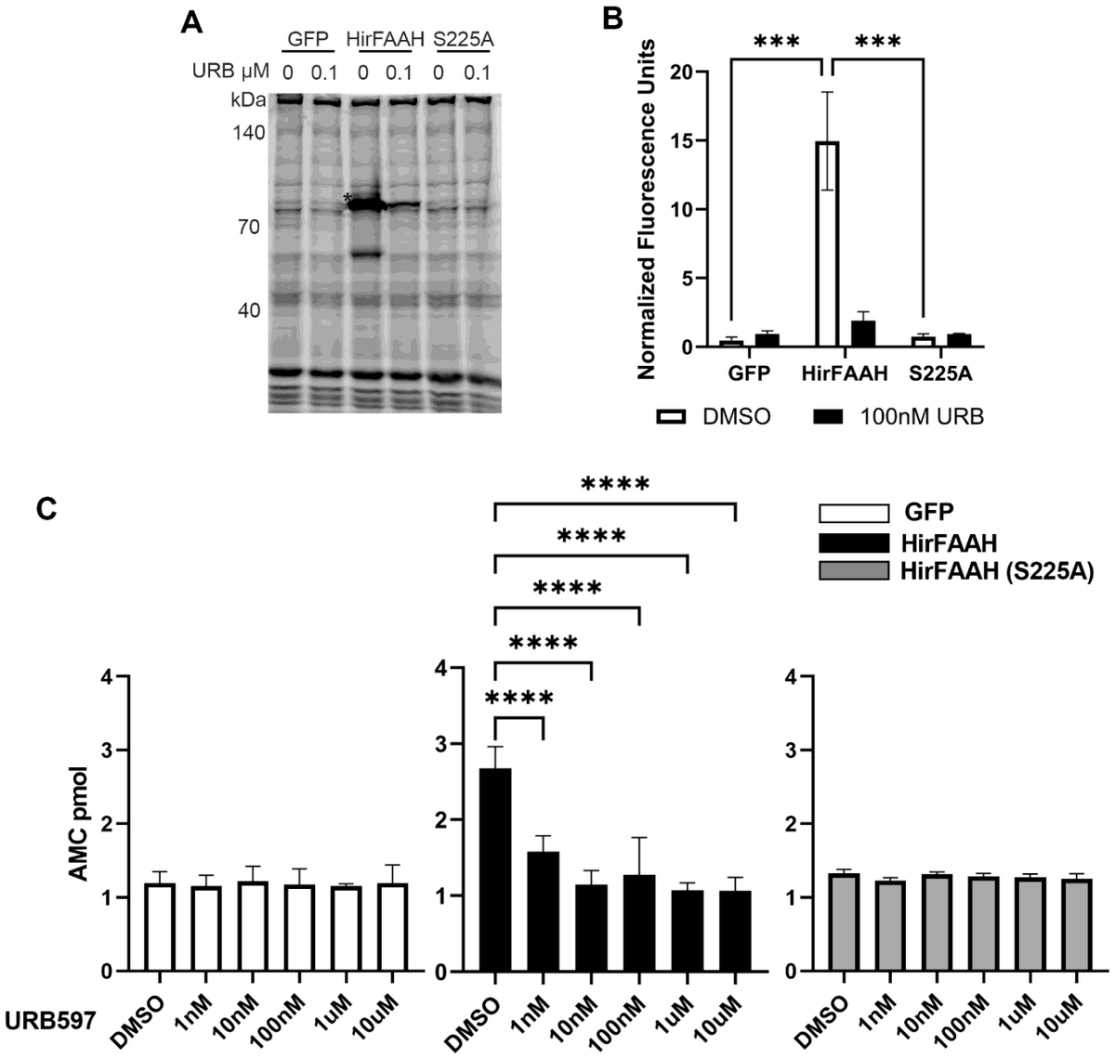
Serine hydrolase and AACA activity of HirFAAH- and mutant HirFAAH-containing microsomes. (a) Representative fluorescent image of TAMRA-FP^™^ labeled microsome fractions from GFP-, HirFAAH-, and HirFAAH(S225A)-transfected cells treated with vehicle (0; 2% DMSO) or 0.1 μM URB597. Asterisk (*) labels presumed FAH in the HirFAAH lanes that is absent in the GFP and S225A lanes. (b) Mean±SE of the HirFAAH band (*) was normalized to the fluorescence signal from the 55kDa band (#) in 6a. The HirFAAH(S225A) mutant exhibited little or no serine hydrolases activity, similar to GFP-containing samplea, when URB597 was omitted (0 nM). 100 nM URB significantly reduced HirFAAH activity, but has no affect on GFP or S225A samples. (c) AAMCA hydrolysis by microsomal fractions isolated from 293HEK transfected lysates. Samples from GFP- and HirFAAH(S225A)-transfected cells exhibit no increase in activity above background and are not affected by increasing concentrations of URB597. HirFAAH exhibited substantial AAMCA activity above background and this activity was inhibited by URB597. Values are the mean and standard error of the mean of a minimum of three independent experiments (*P<0.05, ** P<0.01. *** *P*<0.001 and **** P<0.0001).

In order to maximize the amount of HirFAAH used in the AAMCA assay and increase the assay’s sensitivity, the membrane fraction of HirFAAH was further purified into a microsomal fraction. Previous work has shown that FAAH activity is highest in the microsomal fraction [40]. As a validation step to assess our microsomal isolation process, the ABPP assays were repeated using microsomal fractions from the transfected 293HEK cells. In these experiments, serine hydrolase activity was compared from microsome samples prepared from cells transfected with HirFAAH (N = 5), the GFP-only vector (N = 2), and HirFAAH(S225A) mutant (N = 4). Consistent with our previous experiments using membrane fraction samples (Fig. 5), the ActiveX^™^ TAMRA-FP probe labeled a protein (~ 83kDa) in the HirFAAH-expressing microsomal fractions that was not present in the GFP samples (Fig. 6a, b). In addition, no serine hydrolase activity was observed samples transfected with the HirFAAH(S225A) mutant, indicating that the mutation at the proposed active site did disrupt enzyme function. 100 nM URB597 significantly inhibited HirFAAH activity with no effect on samples from the GFP-only and HirFAAH(S225A) groups. This was confirmed by 2-way ANOVA which showed a significant effect of gene product (F_4,46_ = 9.68, p < 0.005), URB597 treatment (F_4,46_ = 5.36, p < 0.05), and interaction effect (F_4,46_ = 7.38, p < 0.01). To summarize, these TAMRA-FP experiments demonstrated that HirFAAH exhibited the expected serine hydrolase activity, and this activity was reduced on a concentration-dependent manner by the FAAH inhibitor URB597. Furthermore, mutation of HirFAAH at the putative active site did prevent serine hydrolase activity.

Next, microsomal fractions from 293HEK cells expressing GFP (N = 3), HirFAAH (N = 4) or HirFAAH(S225A) (N = 4) were incubated with AAMCA plus either 2% DMSO or increasing concentrations of URB597. Figure 6c shows background levels of AMC production in samples expressing GFP alone incubated in 2% DMSO. No statistically significant changes in AMC levels was observed in GFP-only sample incubated in any of the URB597 concentrations (1nM, 10nM, 100nM, 1μM and 10μM). Samples containing wildtype HirFAAH and incubated in DMSO showed substantial AMC production over background levels indicating HirFAAH specific AAMCA hydrolysis (Fig. 6c), consistent with other FAAHs that metabolize AEA. Additionally, there was a statistically significant decrease in the AMC production when the HirFAAH microsomal fraction was incubated with URB597 (Fig. 6c). Concentrations ranging from 1 nM to 10 μM URB597 produced significant inhibition of enzymatic activity (post hoc p < 0.001). Interestingly, samples in this analysis appeared more sensitive to inhibition by URB597 compared to samples in the ABPP assay. In the AAMCA assay, 1 nM URB597 produced a significant decrease in enzymatic activity, whereas a minimum of 100 nM was required to produce a significant decrease relative to the vehicle control in the ABPP assays (see Fig. 5c vs. 6c). This may be a consequence of different sensitivities between the AAMCA and ABPP assays. In the case of HirFAAH(S225A) samples incubated in DMSO, AMC production were at background levels (Fig. 6c) indicating that no hydrolysis activity in this mutant FAAH, consistent with the site mutation interfering with enzymatic activity. HirFAAH(S225A)-containing samples exhibited no change in AMC production when pre-incubated with increasing concentrations of URB597 (Fig. 6c). Two-way ANOVA showed a significant effect of gene product indicating that only HirFAAH-containing samples exhibited enzymatic activity (F_2,48_ = 12.47, p < 0.001). Analysis also showed a significant effect of URB597 concentration (F_5,48_ = 13.53, p < 0.001) and a significant interaction effect (F10,48 = 13.10, p < 0.001), indicating that the FAAH inhibitor did reduce enzymatic activity of HirFAAH with no effect on the HirFAAH(S225A) mutant or the GFP control.

### Effects of FAAH inhibition on synaptic transmission

**Figure 7:**
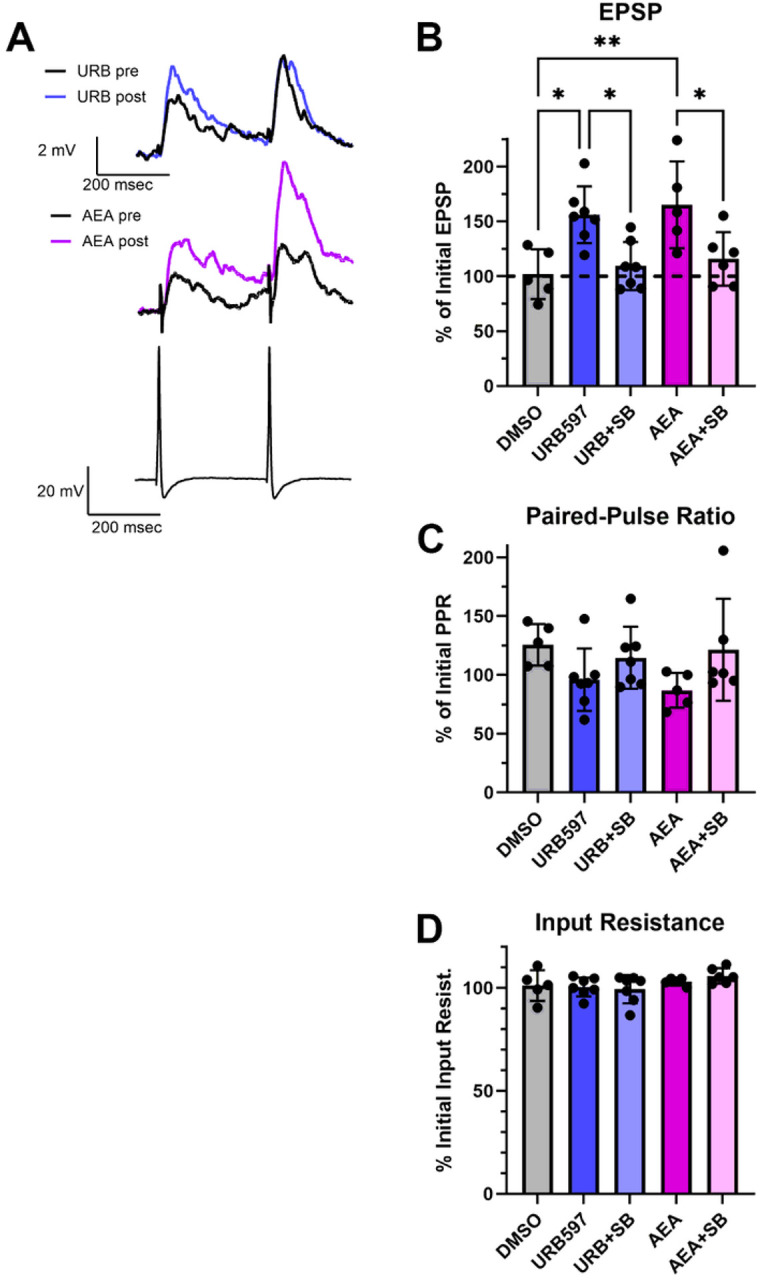
Pharmacological inhibition of FAAH mimics the effects of AEA on synaptic transmission. (a) Sample recordings of paired EPSPs in AP cells before and after 1 μM URB597 (top traces) or 1 μM AEA (middle traces). Bottom traces are a recording of the presynaptic P cell action potentials. (b) Both URB597 and AEA produced significant potentiation that was not observed in the vehicle control experiments (DMSO). Furthermore, the TRPV inhibitor SB366791 (10 μM) blocked potentiation in elicited by URB597 or AEA. (c) No changes in paired-pulse facilitation ratio (PPR) were observed in any of the experimental groups. (d) No changes in paired-pulse input resistance were observed in any of the experimental groups.

Next, we wanted to examine whether URB597 has functional effects on the *Hirudo* CNS consistent with inhibition of FAAH. Therefore, we examined the effects of URB597 on synaptic transmission by pressure sensory neurons (P cells) in acutely isolated *Hirudo* ganglia. In previous studies, a 15 min AEA application was found to produce long-lasting (1 hour) in P cell synapses [24, 41]. If URB597 is increasing AEA levels by inhibiting HirFAAH, then one would expect URB597 to mimic the effect of exogenously applied AEA. EPSP amplitude was recorded prior to and then 1 hour following a 15 min application of 1 μM URB597 (N = 7) since this was a concentration that appeared to effectively inhibit HirFAAH activity (see Figs. 5c and 6c). Experiments using 1 μM AEA (N = 5) were also conducted in parallel to compare to the URB597 results. The FAAH inhibitor did significantly potentiate the P cell EPSP compared to the 0.001% DMSO control group (N = 5) and did so at a level similar to AEA (Fig. 7a, b). One-way ANOVA showed a significant effect of treatment group (F_4,25_ = 6.55, p < 0.0001), with the AEA- and URB597-treated ganglia exhibiting significant potentiation compared to the DMSO control groups (p < 0.05 and < 0.01, respectively). In past experiments, synaptic potentiation by AEA was blocked by the TRPV inhibitor SB366791, indicating that AEA acted on a Hirudo TRPV-like channel [24]. Here the ability of 10μM SB366791 to block synaptic potentiation by AEA was confirmed (Fig. 6B; AEA + SB group, N = 6; p < 0.05). Furthermore, the co-application of the TRPV inhibitor with URB597 also blocked the FAAH inhibitor’s capacity to produce synaptic potentiation (URB + SB, N = 7; p < 0.05).

Consistent with prior studies, AEA-induced synaptic potentiation produced no change in paired-pulse facilitation ratio (PPR) compared to the DMSO controls (Fig. 7c). This suggests that synaptic potentiation occurs at the post-synaptic level. As with AEA, URB597 also did not significantly change PPR. One-way ANOVA confirmed no significant difference in the percent change in PPR across an of the treatment groups (F_4,25_ = 1.93, p > 0.05). For all the treatment groups, no change was observed in the input resistance of the postsynaptic cell (Fig. 7d), indicating that the observed increases in EPSP amplitude were not due to changes in the intrinsic excitability of the postsynaptic neurons, at least as measured in the soma (F_4,25_ = 1.42, p < 0.05).

## Discussion

In this study we identified and characterized putative FAAH-encoding gene from the *Hirudo* transcriptome. The sequence identified was amplified from the *Hirudo* CNS and codes for a full-length, functional FAAH protein. Initial sequence alignment of the HirFAAH indicated the greatest similarity to human FAAH-2 isoform. Further comparisons with other FAAH-2 or putative FAAH-2 sequences from a range of vertebrate and invertebrate species supported the conclusion that HirFAAH is a FAAH-2 orthologue. Most invertebrates appear to only possess FAAH-2 isoforms [42] and our database analysis of other invertebrate FAAH’s would seem to confirm this. One notable exception is in C. elegans where a recent study actually reported six FAAH isoforms [21], including what appear to be FAAH-1 and FAAH-2 orthologues. It is unclear whether this expansion of FAAH-encoding genes is limited to C. elegans, others in the phylum Nematoda, other ecdysozoan phyla (e.g., Arthropoda), or other invertebrate in general. Similarly, it is unclear whether the FAAH-1 gene in C. elegans represents a conserved orthologue to vertebrate FAAH-1 or is an example of convergent evolution. Across the vertebrate phyla FAAH-1 and − 2 have a somewhat unusual distribution. Both FAAH-1 and − 2 are present in amphibians, fish, non-placental mammals, and placental primates. However, FAAH-2 appears to be absent in non-primate placental mammals [37].

Human FAAH-1 and − 2 differ in their catalytic capacity, with FAAH-1 having higher rates of activity [33, 35]. Another distinction is that while FAAH-1 and − 2 are both membrane bound proteins, they have different orientations with the membrane. FAAH-1 is oriented with its catalytic region facing the cytoplasm while FAAH-2 is oriented facing the luminal compartment of the endoplasmic reticulum [33]. FAAH-2 may also be incorporated into lipid droplets within the cytoplasmic compartment [35]. In that study, immunofluorescence microscopy showed HsaFAAH2 localized to ring-like structures in the cytoplasm found to be lipid droplets. HirFAAH protein was also found to localized to ring-like structures in the cytoplasm that appeared to be similar lipid droplets.

That HirFAAH is a functional serine hydrolase was confirmed by its activity in the ABPP assay with the ActveX^™^ TAMRA-FP probe. The TAMRA-FP probe labeled many proteins when incubated with either lysates or membrane fractions from transfected 293HEK cells, however, only one band differed between the GFP-only vector control and HirFAAH expressing cells. Furthermore, the protein differentially labeled by TAMRA-FP corresponded to the predicted molecular weight for the GFP-tagged HirFAAH. The enzymatic activity of HirFAAH was further elucidated using the AAMCA-based fluorescence assay that measures the hydrolysis of AAMCA to generate arachidonic acid and the highly fluorescent AMC. Together, these experiments provide two independent assessments of serine hydrolase activity by HirFAAH. As an additional test of HirFAAH biochemical function, the critical catalytic site for serine hydrolase activity was mutated (serine to alanine) to presumably inactivate the protein. In the TAMRA-FP assay of serine hydrolase activity, fluorescence at the band corresponding with hirFAAH was reduced to background levels in the HirFAAH(S225A) sample. Similarly, the fluorescent signal produced when HirFAAH(S225A) samples is incubated with AAMCA was also significantly lowered to background levels.

URB597 is a widely used FAAH inhibitor that is a potent blocker of FAAH-1 and − 2 with IC_50_ of approximately 100 nM and 5 nM, respectively [33]. Similar concentration dependent inhibition of HirFAAH by URB597 was observed in both the TAMRA-FP and AAMCA-based fluorescence assays. Lower concentrations of URB597 were effective in the AAMCA assay compared to the ABPP assy. This may be due to different sensitivities of the two assays or because the AAMCA assay was carried out in microsome fractions that may have more concentrated amounts of FAAH per sample. To our knowledge this is only the second report in which URB597 has been validated to inhibit FAAH in an invertebrate, the other being in the FAAH-2 isoform in C. elegans [21]. This represents an important validation for using URB597 in future studies in Hirudo focusing on the neurobehavioral effects of AEA.

Given that URB597-blocking of FAAH presumably increases AEA levels, we examined whether this drug mimicked the synaptic effects of exogenously applied AEA. In P cell synapses, AEA elicits persistent potentiation and similar increases in the P-to-AP EPSP were observed following URB597 treatment. Furthermore, the URB597-induced potentiation was blocked by co-treatment with the TRPV inhibitor SB366791, again consistent with the effects of AEA which are also appears to be mediated by Hirudo TRPV-like receptor [24, 41]. It is well-established that TRPV1 acts as a cannabinoid receptor in mammals [43, 44] and our studies to date suggest that a TRPV like channel mediates the effects of both 2-AG and AEA in Hirudo [23, 41, 45]. Neither AEA nor URB597 elicited a change in PPR, indicating the site of potentiation was postsynaptic. This agrees with our previous studies of AEA modulation of the P synapses which lacks TRPV-like channels. Potentiation of P synapses is mediated first by a depression of tonic inhibition of the P cell by AEA [46]. The resulting cannabinoid-mediated disinhibition “gates” synaptic potentiation via a mechanism that is dependent on NMDA receptor activation and CamKII [47].

Endocannabinoid signaling in general, and AEA in particular, play an important role in a variety of behavioral and physiological processes across the animal kingdom. FAAH plays an important role in regulating AEA levels and represents a “druggable” site for potentially raising AEA levels to produce a clinical effect, e.g., treating chronic pain. In preclinical studies, increasing AEA levels via pharmacological inhibitors or genetic knockdown of FAAH has been shown to produce analgesic effects [48, 49]. This approach is further supported by a single clinical case of chronic insensitivity to pain that involves a mutation reducing FAAH activity and raising AEA levels [50]. However, an attempt to develop an effective FAAH inhibitor (PF-04457845) as a treatment for chronic pain was unsuccessful due to lack of efficacy [51]. There are undoubtably many reasons for this disconnect between preclinical studies and clinical results. However, one element that we have focused on is that endocannabinoids can have both pro- and anti-nociceptive effects, a finding supported by our work in Hirudo and a number of other findings in mammals [12, 52, 53]. In *Hirudo* we have found the both 2-AG and AEA depress nociceptive synapses, producing an anti-nociceptive effect, but disinhibit/potentiate non-nociceptive synapses, producing a pro-nociceptive effect [24, 27, 46, 47]. Similar observations have also been observed in the mammalian spinal circuitry [54, 55]. In *Hirudo*, different patterns of afferent activity elicit either the pro- or anti-nociceptive effects of endocannabinoids [24, 25, 27, 56]. An approach we plan to investigate in the future is whether pairing FAAH inhibition, e.g., URB597, with certain patterns of activity that elicit endocannabinoid synthesis, e.g., repetitive non-nociceptive afferent stimulation, can selectively produce anti-nociceptive effects. The findings from these studies may inform how best to deploy FAAH inhibitors in the clinical setting as a treatment for chronic pain.

## Figures and Tables

**Table 1:** Primer design

Purpose	Primer Sequence
HirFAAH cloning into pcDNA3-EGFP	5’-CCCGATATCATGAAAAAAATGATGATTGATAGC-3’
	5’-AGGATCAAGTTAGTTAAAATTGCGGCCGCACA-3’
Site directed mutagenesis HirFAAH S225A	5’-AGGAGCAGACATAGGTGGAGCCATTAGAATGCC-3’
	5’-TCCACCTATGTCTGCTCCTACTCCAAATGA-3’
hirFAAH qRT-PCR	5’-GCGAGTGGTTTTACCTTGCC-3’
	5’-TCTACCGCCTCCCTTGACTT-3’
R60S qRT-PCR	5’-AGTGGCAACTTTGGATTTGG-3’
	5’-TTTGACAGCCTCTTCCTTGG-3’

## Data Availability

Data available on request
